# Recurrent Cavus Foot in an Adolescent With Marfan Syndrome: A Case Report

**DOI:** 10.7759/cureus.94448

**Published:** 2025-10-13

**Authors:** Abdulrahman M Alqahtani, Abdulaziz Bayounis, Nawaf Alamri, Mishary Aldakhil, Nawaf Alogayyel

**Affiliations:** 1 Department of Surgery, Division of Orthopedic Surgery, King Abdulaziz Medical City, Riyadh, SAU; 2 Department of Pediatric Orthopedics, King Abdulaziz Medical City, Riyadh, SAU; 3 Department of Pediatric Orthopedics, King Abdullah Specialized Children Hospital, Riyadh, SAU; 4 Department of Orthopedic Surgery, King Abdulaziz Medical City, Riyadh, SAU

**Keywords:** cavovarus foot, cavus foot, marfan syndrome, pes planus (flatfoot), recurrent cavovarus foot

## Abstract

Patients with Marfan syndrome typically have pes planus foot deformity, while some develop the opposite, cavus foot. Here, we describe the case of an adolescent with Marfan characteristics and a painful cavovarus foot, who underwent surgical correction but later experienced recurrent deformity and metatarsalgia. Osteotomies are the preferred treatment for cavus foot in patients aged five through skeletal maturity, providing symptom relief and preserving future definitive surgical options. In contrast, joint arthrodesis procedures are reserved for those who have reached skeletal maturity. In this case report, a 13-year-old male with Marfan syndrome underwent joint-sparing osteotomies at 12 for severe cavovarus deformity, achieving deformity correction and pain relief for two years until symptoms and deformity recurred. He was offered further corrective surgery, but his family remained hesitant. This case highlights that, while joint-sparing procedures before skeletal maturity offer temporary relief and preserve future options, recurrence is a possibility; therefore, patients and their families should be counseled accordingly.

## Introduction

Marfan syndrome is one of the most common inherited connective tissue disorders. It follows an autosomal dominant inheritance pattern and affects approximately 1 in every 3,000 to 5,000 people [1,2]. The condition is caused by mutations in the *FBN1* gene located on chromosome 15, which is responsible for producing fibrillin, a key protein that helps give connective tissue its strength and elasticity [3,4].

Individuals with Marfan syndrome can have a wide range of symptoms, ranging from just a few mild signs to severe cases that appear early in life and affect multiple organs [5]. The most typical features involve the eyes, heart and blood vessels, and skeleton, but sometimes the lungs, skin, and nervous system can also be involved [6,7].

One of the most serious complications of Marfan syndrome is related to the aorta, the main artery of the body. Issues such as aortic root enlargement and aortic dissection are the leading causes of reduced life expectancy in affected individuals [8].

Regarding foot deformity, usually Marfan Syndrome patients present with pes planus foot deformity [9]. These foot deformities can range from mild and asymptomatic to more severe forms. Interestingly, while flatfeet (pes planus) are more commonly associated with Marfan syndrome, some individuals present with the opposite problem, a cavus foot [10].

## Case presentation

A 13-year-old boy, a known case of Marfan syndrome, had a prior medical history including mild scoliosis and pectus carinatum, both of which were managed with observation and follow-up, with no significant past surgical history. At the age of 10, the patient presented to the orthopedic clinic with a bilateral cavovarus foot, with the right being worse than the left. A trial of physiotherapy (stretching and strengthening exercises for the Achilles tendon and quadriceps muscles) with an antivarus shoe failed. During follow-up, there was no improvement, and the deformity was worsening over time, with the patient feeling pain affecting his daily activities.

**Figure 1:**
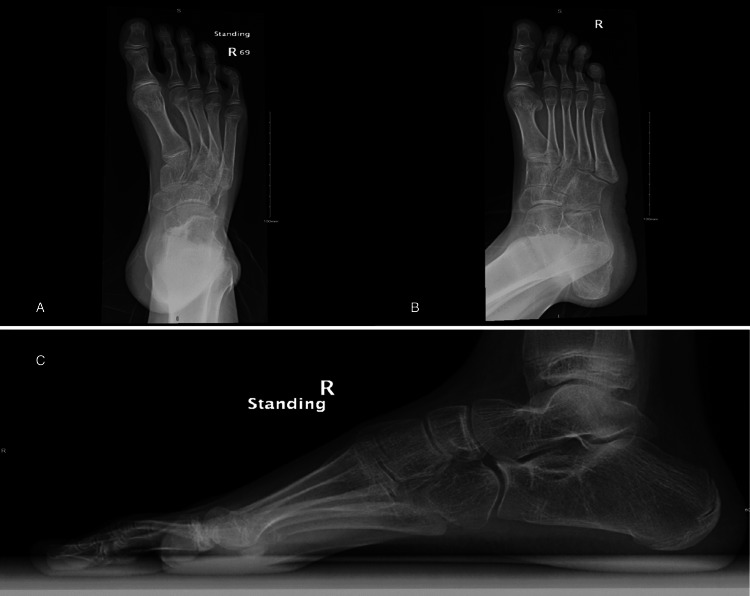
Preoperative anterior posterior and lateral weight-bearing and mortise views revealing cavovarus deformity. The anteroposterior view shows varus deformity with metatarsal overlap (A). The oblique view shows no coalition (B). The lateral view reveals an increase in Meary’s angle (C).

**Figure 2:**
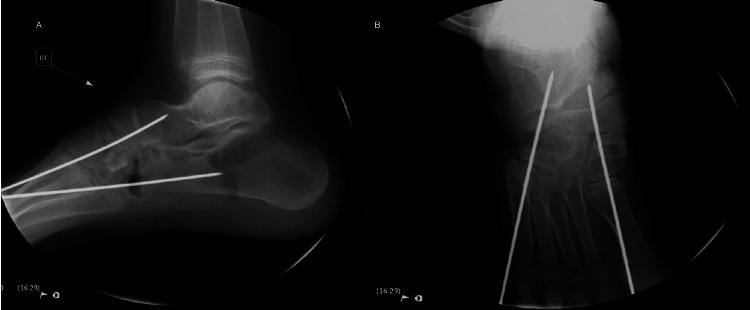
Intraoperative imaging showing medial cuneiform osteotomy and cuboid osteotomy with K-wire fixation. The intraoperative lateral view shows a decrease in Meary’s angle compared to the preoperative lateral view (A). The anteroposterior view shows the corrected forefoot abduction (B).

**Figure 3:**
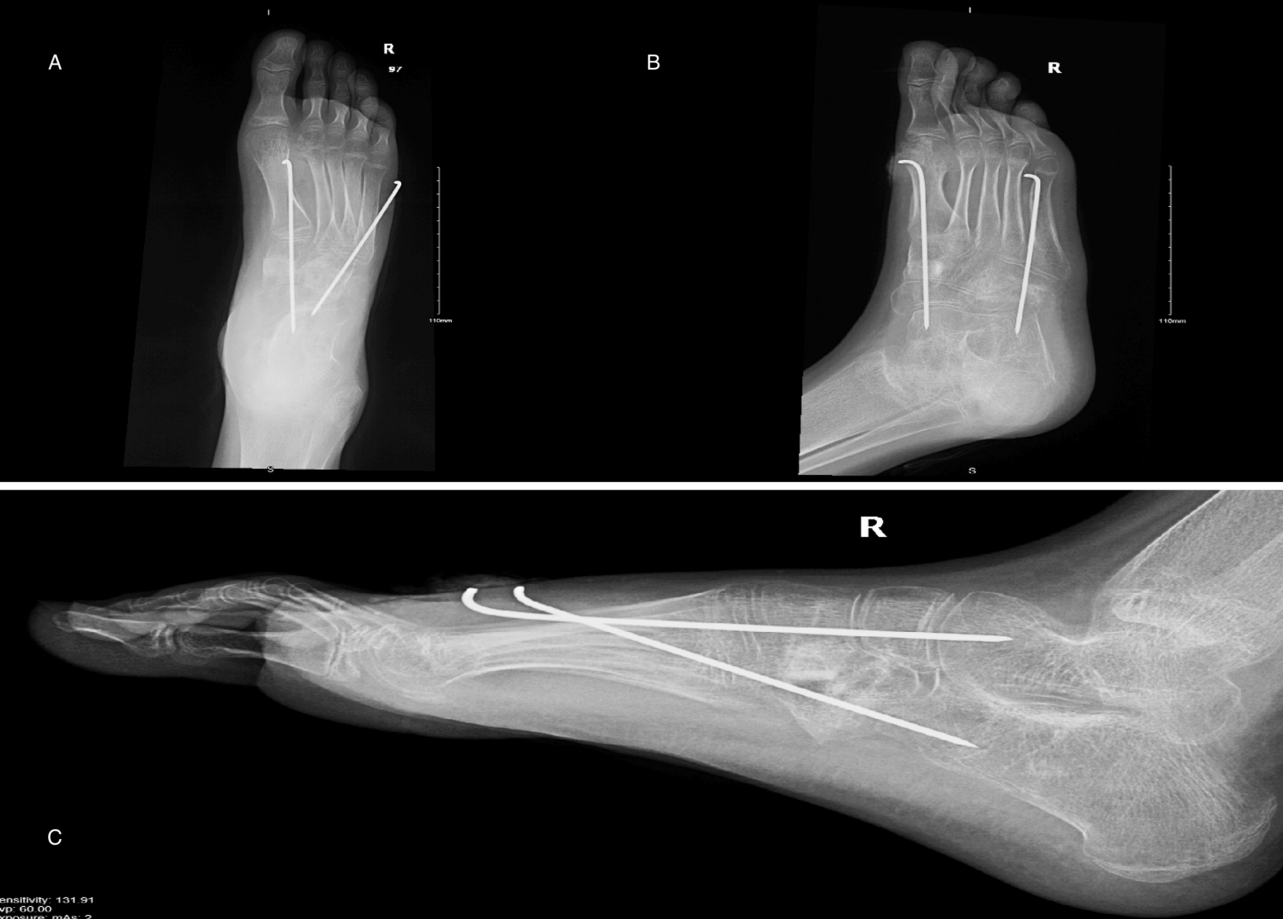
Postoperative week six anteroposterior, lateral, and oblique views showing medial cuneiform osteotomy and cuboid osteotomy with the K-wires in place. The anteroposterior and oblique view show medial cuneiform and cuboid osteotomy with ongoing healing and maintained alignment (A, B). The lateral view reveals a maintained Meary’s angle (C).

**Figure 4:**
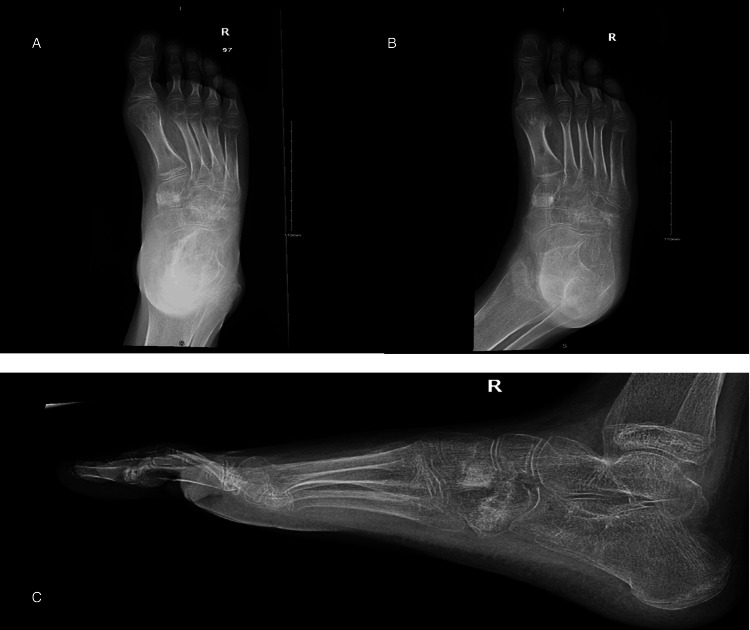
Postoperative week eight anteroposterior, lateral, and oblique views showing maintained foot correction after the removal of the K-wires. The anteroposterior and the oblique view show medial cuneiform and cuboid healed osteotomy and maintained alignment (A, B). The lateral view reveals the same Meary’s angle as postoperative week six (C).

**Figure 5:**
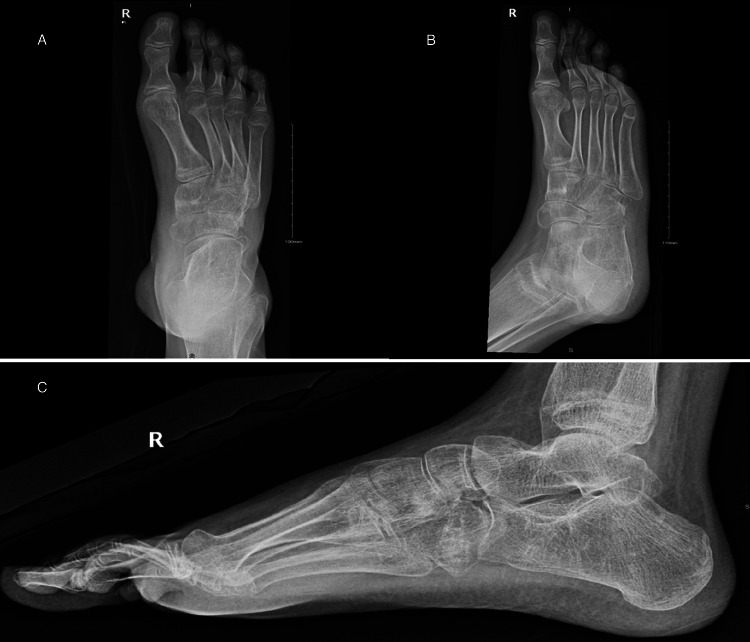
Postoperative four months anteroposterior, lateral, and oblique views showing recurred cavovarus foot deformity. The anteroposterior view shows the varus deformity with a metatarsal overlap (A). The oblique view shows healed osteotomy sites (B). The lateral view reveals increased cavus deformity, represented by an increase in Meary’s angle (C).

Additionally, the patient complained of a bilateral intoeing gait and multiple falls (Figure 1). At the age of 12, surgical intervention was decided and agreed with the family to start with the right foot. The procedure included plantar fascia release, tibialis posterior Z-lengthening, release of the talonavicular joint, medial cuneiform wedge open wedge osteotomy, peroneus longus transfer to peroneus brevis, and cuboid closed wedge osteotomy (Figure 2). Weight-bearing was initiated in the clinic after six weeks, following the healing of the osteotomy and removal of the K-wire (Figure 3). After a four- and seven-month follow-up, the patient was happy, pain was relieved, and the foot was plantigrade and corrected with mild valgus during examination. Imaging showed satisfactory correction (Figures 4, 5). At a one-year follow-up, the patient came back with recurrent varus deformity of the right foot and flexible hind foot. The patient was offered another corrective surgery, but the patient and the family needed time to think about it.

## Discussion

To our knowledge, only two case reports in the literature have described Marfan syndrome associated with cavus foot deformity [11,12]. As with all cases of cavus foot, it is essential to rule out underlying neurological causes; hence, we referred our patient to neurology for further evaluation. The neurological examination and spinal imaging were unremarkable, with no signs suggesting a neurogenic origin.

In reviewing the literature for the prognosis of surgical correction in Marfan patients with cavus foot, we found only one detailed case report. In that case, the patient underwent surgery at age nine, which included gastrocnemius lengthening, Dwyer calcaneal osteotomy, cuboid closing osteotomy, medial cuneiform plantar-based opening wedge osteotomy, V-to-Y skin plasty, and capsulotomy with pinning of the fourth and fifth metatarsophalangeal joints. The procedure provided eight years of symptom relief, but due to the underlying connective tissue disorder, the deformity eventually recurred and progressed.

In our case, the postoperative results were initially satisfactory, both in terms of pain relief and the position and alignment of the foot, as confirmed by postoperative X-rays. The foot was plantigrade with mild valgus, and both the calcaneal pitch and Meary’s angle had improved. However, during follow-up, the patient unfortunately developed a recurrence of the deformity, including a return of the varus alignment.

Recurrence of cavus foot deformity following surgical correction in patients with Marfan syndrome can be attributed to several interrelated factors, primarily rooted in the underlying connective tissue abnormality. Marfan syndrome is caused by mutations in the *FBN1* gene, which leads to defective fibrillin-1 and results in weakened connective tissues and ligamentous laxity [13]. This inherent tissue instability reduces the long-term effectiveness of surgical corrections and predisposes to the gradual recurrence of deformities. Additionally, the progressive skeletal overgrowth characteristic of Marfan syndrome, especially during adolescence, can contribute to the reemergence of foot malalignment despite initially successful procedures [14].

Moreover, suboptimal soft tissue healing and altered mechanical loading due to muscle imbalance may further undermine structural corrections [15]. In some cases, incomplete correction or failure to address the multi-planar nature of the deformity (hindfoot, midfoot, and forefoot) can also lead to recurrence [16]. Finally, although rare, neuromuscular etiologies should be carefully excluded, as they can coexist and compound the deformity.

## Conclusions

Cavus foot is a rare but possible feature of Marfan syndrome. While surgery can improve foot alignment and relieve symptoms, there is always a risk that the deformity will come back over time. This is mostly due to the soft, stretchy connective tissue that is typical in Marfan patients. Before surgery, it is important to rule out any underlying neurological causes and to set realistic expectations with the patient and their family. Regular follow-up is key to detecting any recurrence early and adjusting treatment as needed.
